# Effectiveness of Structured Education Programmes on Diet and Exercise for Preventing Type 2 Diabetes in Adults With Prediabetes: A Systematic Review

**DOI:** 10.7759/cureus.114859

**Published:** 2026-08-20

**Authors:** Chioma G Muoghalu, Geraldine Ifeoma Nwafor, Chidinma Onyegbule, Cosmas C Ofoegbu, Ndianabasi E Ekong, John Ohiri, Godwin Abraham, Danvictor A Ebirim

**Affiliations:** 1 Medicine, University of Galway, Galway, IRL; 2 Medicine, University Hospital, Waterford, IRL; 3 Family Medicine, Alex Ekwueme Federal University Teaching Hospital, Abakiliki, NGA; 4 Community and Family Medicine, Fort Saint John Hospital, Fort Saint John, CAN; 5 Health Sciences, Central Washington College Enugu, Enugu, NGA; 6 Public Health and Health Systems Management, Akwa Ibom State College of Education, Afaha Nsit, NGA; 7 Chemical Pathology, Federal University of Technology, Owerri, Owerri, NGA; 8 Oncology, Midland Metropolitan University Hospital, Birmingham, GBR; 9 Medicine, Federal University Teaching Hospital, Owerri, NGA

**Keywords:** diet, exercise, prediabetes, structured education, type 2 diabetes

## Abstract

Diet and exercise can prevent or delay progression from prediabetes to type 2 diabetes mellitus, but lifestyle advice in routine care may be brief, inconsistent, or unstructured. This systematic review assessed whether structured education programmes on diet and exercise are more effective than usual care, standard advice or less intensive lifestyle intervention in preventing or delaying type 2 diabetes among adults with prediabetes.

The review was conducted in accordance with Preferred Reporting Items for Systematic Reviews and Meta-Analyses (PRISMA) guidance and registered in PROSPERO (CRD42023484038). CINAHL (Cumulative Index to Nursing and Allied Health Literature), Embase, Ovid MEDLINE, Web of Science and Google Scholar were searched for English-language studies from database inception to July 2026. Randomised and cluster-randomised controlled trials involving adults with prediabetes, impaired glucose tolerance, or impaired fasting glucose were included if they compared structured education focused on diet, exercise or both with usual care, standard advice or a less intensive intervention. Data were synthesised narratively. Eighteen randomised or cluster-randomised controlled trials met the inclusion criteria.

Most studies reported favourable effects of structured education on diabetes incidence, glycaemic indices and weight-related outcomes compared with usual care or less intensive lifestyle advice, while effects on cardiovascular risk markers were less consistent. Structured education on diet and exercise appears more effective than usual care or less intensive lifestyle advice in preventing or delaying type 2 diabetes among adults with prediabetes.

## Introduction and background

Prediabetes is an intermediate metabolic state in which blood glucose levels are higher than normal but below the diagnostic threshold for type 2 diabetes mellitus. It includes impaired fasting glucose, impaired glucose tolerance and elevated glycated haemoglobin within the non-diabetic hyperglycaemic range [1-3]. Although not all individuals with prediabetes progress to diabetes, the condition is associated with increased risk of type 2 diabetes, cardiovascular disease and other cardiometabolic abnormalities, including obesity, hypertension and dyslipidaemia [4,5].

The global burden of diabetes continues to rise, making prevention a major public health priority. The International Diabetes Federation estimated that 589 million adults aged 20-79 years were living with diabetes in 2024, with this number projected to increase to 853 million by 2050 [6]. Prediabetes represents an important opportunity for early intervention because progression to type 2 diabetes can be delayed or prevented when appropriate lifestyle measures are implemented. Early identification of adults with prediabetes and effective support for lifestyle change are therefore essential components of diabetes prevention.

Dietary modification and increased physical activity are well-established strategies for reducing the risk of progression from prediabetes to type 2 diabetes. Landmark diabetes prevention trials have shown that lifestyle interventions involving diet, exercise and weight reduction can significantly reduce the incidence of type 2 diabetes among high-risk adults [7-9]. These findings are supported by systematic reviews showing that diet, physical activity or combined lifestyle interventions can reduce diabetes incidence and improve glycaemic and weight-related outcomes in people at increased risk of type 2 diabetes [10,11]. However, in routine clinical practice, lifestyle advice is often delivered briefly, inconsistently or without structured follow-up. As a result, patients may understand that diet and exercise are important but may not receive adequate support to translate this knowledge into sustained behavioural change.

Structured education programmes provide a more organised approach to lifestyle intervention. Unlike brief or unstructured advice, structured programmes usually include planned educational content, goal-setting, behavioural counselling, self-management support, follow-up contacts and reinforcement of dietary and physical activity recommendations. These programmes may be delivered individually or in groups, in primary care or community settings, or through technology-supported platforms. The aim is not only to provide information but also to improve motivation, self-efficacy and adherence to lifestyle recommendations.

Structured education has become an important part of diabetes care, with programmes such as Diabetes Education and Self-Management for Ongoing and Newly Diagnosed people supporting self-management among individuals with type 2 diabetes [12,13]. Similar approaches have been adapted for adults with prediabetes, including programmes focused on walking, physical activity promotion, dietary change, weight loss and combined lifestyle modification [14,15]. These interventions vary in intensity, duration, personnel involved and mode of delivery, but they share a common purpose: to provide systematic support for lifestyle change rather than relying on general advice alone.

Despite the established benefit of diet and exercise, the additional value of delivering these interventions through structured education remains important to clarify. Landmark trials reported significant reductions in progression to type 2 diabetes following structured lifestyle interventions [7-9], whereas the pragmatic community-based Let’s Prevent Diabetes trial reported a more modest, non-significant reduction in diabetes risk [15]. Differences in intervention intensity, frequency of contact, follow-up duration and comparator groups make direct comparison difficult. A systematic synthesis is therefore needed to determine whether structured education programmes on diet and exercise are more effective than usual care, standard advice or less intensive lifestyle counselling in adults with prediabetes.

This systematic review aimed to assess the effectiveness of structured education programmes on diet and exercise for preventing or delaying type 2 diabetes in adults with prediabetes. The primary outcome was progression to type 2 diabetes, while secondary outcomes included glycaemic control, weight reduction, and cardiovascular risk markers.

## Review

Objective

This systematic review aimed to assess whether structured education programmes on diet and exercise are more effective than usual care, standard advice or less intensive lifestyle intervention in preventing or delaying type 2 diabetes mellitus in adults with prediabetes.

The primary outcome was progression to type 2 diabetes mellitus. Secondary outcomes included glycaemic control, weight reduction and cardiovascular risk markers.

Methodology

Protocol and Registration

This systematic review was conducted in accordance with the Preferred Reporting Items for Systematic Reviews and Meta-Analyses (PRISMA) statement [16]. The protocol was registered prospectively in the International Prospective Register of Systematic Reviews (PROSPERO; registration number: CRD42023484038).

Search Strategy

An electronic literature search was conducted in CINAHL (Cumulative Index to Nursing and Allied Health Literature), Embase, Ovid MEDLINE, Web of Science and Google Scholar (first 10 pages according to relevance) for studies published in English from database inception to July 2026. The reference list of included studies was also manually assessed for eligible results. The principal search strategy was:

(prediabetes OR “pre-diabetes” OR “impaired glucose tolerance” OR “impaired fasting glucose”) AND (“structured education” OR “patient education” OR “health education” OR “lifestyle education”) AND (diet OR nutrition OR exercise OR “physical activity”) AND (“type 2 diabetes” OR “diabetes prevention” OR “progression to diabetes”)

Search terms were adapted, where appropriate, to meet the indexing requirements of individual databases.

Eligibility Criteria

For this review, structured education was operationally defined as a protocolised intervention with predefined educational or counselling content focused on diet and/or physical activity, explicit lifestyle-related targets, and at least one active behaviour-change or self-management technique, such as goal-setting, action planning, self-monitoring, problem-solving or motivational counselling. Brief general advice or provision of an information leaflet alone, without structured content and an active behaviour-change component, was not eligible.

Randomised and cluster-randomised controlled trials were eligible for inclusion if they involved adults aged 18 years and above with prediabetes, impaired glucose tolerance, or impaired fasting glucose. Eligible interventions were structured education programmes, structured counselling or structured behavioural support focused on diet, exercise or both. Eligible comparators included usual care, standard care, brief advice, minimal intervention or less intensive lifestyle advice.

Studies were excluded if participants had established type 2 diabetes mellitus at baseline, were pregnant, were children or adolescents, or if the intervention was pharmacological without a clearly separable lifestyle education component. Non-randomised studies, observational studies, reviews, editorials, conference abstracts without sufficient outcome data and non-English publications were also excluded.

Study Selection

All retrieved records were exported to Mendeley (Elsevier, London, UK), where duplicates were removed. Titles and abstracts were screened independently by two reviewers against the eligibility criteria. Full texts of potentially eligible studies were then assessed for final inclusion. Disagreements were resolved by discussion and consensus. The selection process was summarised using a PRISMA flow diagram.

Data Extraction

Data were extracted using a standardised data extraction form. Extracted information included author, year of publication, country, sample size, participant characteristics, intervention content, comparator, duration of follow-up and main outcome focus. Data extraction was performed by one reviewer and cross-checked by a second reviewer.

Risk of Bias and Certainty of Evidence

Risk of bias in the included randomised trials was assessed using the Cochrane RoB 2 tool [17]. For cluster-randomised trials, the RoB 2 tool for cluster-randomised designs was applied where appropriate. Studies were judged as having low risk of bias, some concerns or high risk of bias across the domains of randomisation process, deviations from intended interventions, missing outcome data, measurement of outcomes and selection of the reported result. The certainty of evidence for each prespecified outcome was independently assessed by two reviewers using the Grading of Recommendations Assessment, Development and Evaluation (GRADE) approach [18]. Evidence from randomised and cluster-randomised controlled trials was initially considered to be of high certainty and was downgraded where appropriate after considering risk of bias, inconsistency, indirectness, imprecision and publication bias. The certainty of evidence was classified as high, moderate, low or very low. Disagreements were resolved through discussion and consensus.

Data Synthesis

Findings were synthesised narratively using guidance on the conduct of narrative synthesis in systematic reviews [19]. Results were presented according to the primary outcome of progression to type 2 diabetes mellitus and secondary outcomes including glycaemic control, weight reduction and cardiovascular risk markers.

Results

Study Selection

**Figure 1:**
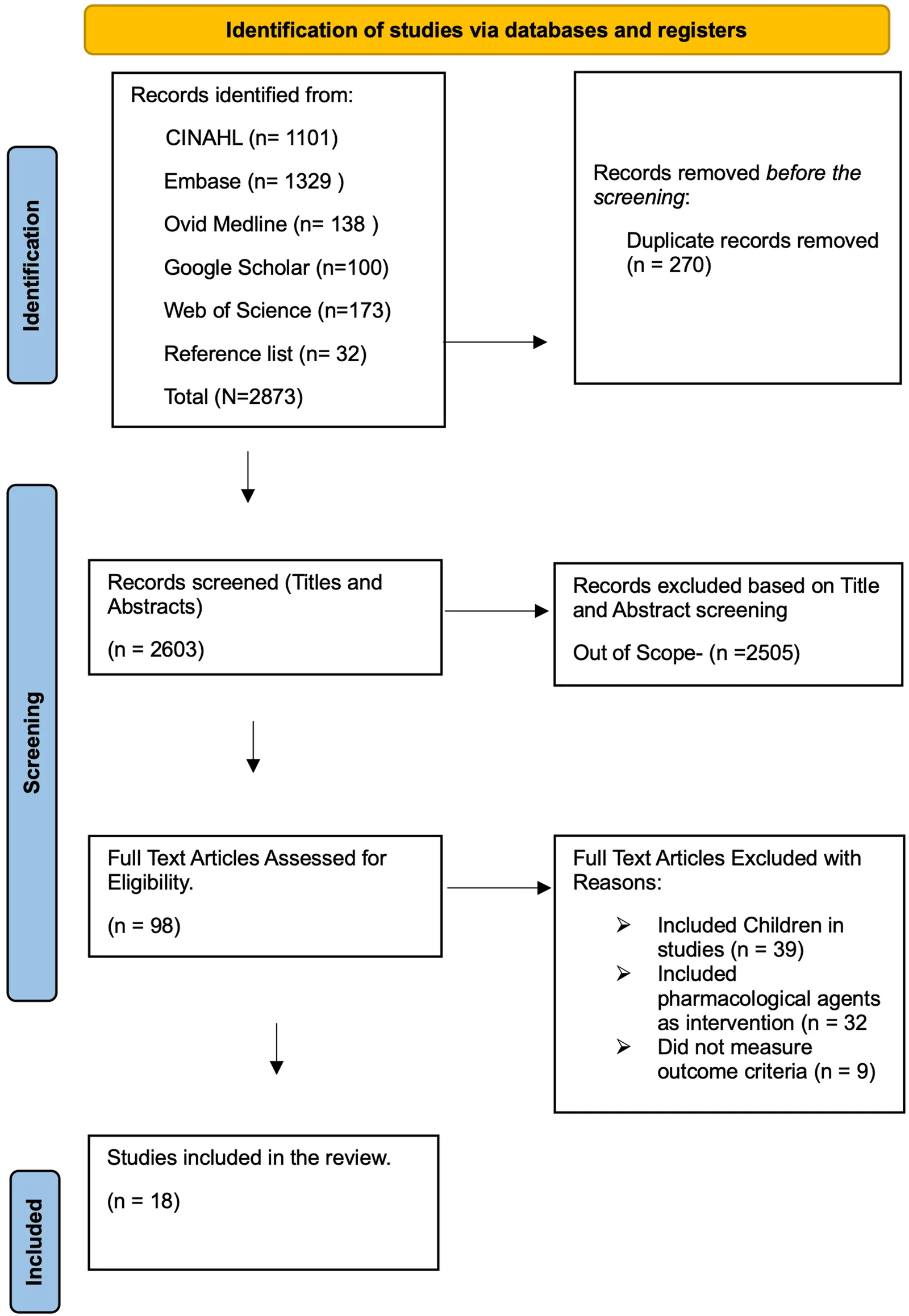
PRISMA flow diagram for study selection PRISMA, Preferred Reporting Items for Systematic Reviews and Meta-Analyses

The searches identified 2873 records: CINAHL 1101, Embase 1329, Ovid MEDLINE 138, Web of Science 173, Google Scholar 100 and reference-list searching 32. After removal of 270 duplicates, 2603 records underwent title and abstract screening, of which 2505 were excluded. After removal of duplicates, title and abstract screening, and full-text assessment, 18 randomised or cluster-randomised controlled trials met the eligibility criteria and were included in this systematic review [7-9,14,15,20-32] (Figure 1). The included studies assessed structured education, structured lifestyle counselling, or structured behavioural support focused on diet, exercise, or both among adults with prediabetes, impaired glucose tolerance, or impaired fasting glucose. Studies were excluded if they involved non-randomised designs, paediatric or pregnant populations, established type 2 diabetes mellitus at baseline, or pharmacological interventions without a clearly separable lifestyle education component.

Characteristics of Included Studies

The 18 included studies were published between 1997 and 2021 and were conducted in China, Finland, the United States, Japan, India, the United Kingdom, the Netherlands, Thailand and Saudi Arabia [7-9,14,15,20-32] (Table 1). The trials varied in setting, intervention intensity, mode of delivery and follow-up duration. Earlier efficacy trials were generally delivered in research or clinical settings, while later trials included primary-care, community-based, hospital-based and structured group-education approaches.

**Table 1:** Characteristics of included studies DPP, Diabetes Prevention Project

Study/Year	Country	Sample Size	Population	Structured intervention	Comparator	Follow-up	Main outcome focus
Pan et al. 1997 [7]	China	577	Adults with impaired glucose tolerance	Structured diet, exercise, or diet-plus-exercise intervention	Usual care	6 years	Diabetes incidence
Tuomilehto et al. 2001 [8]	Finland	522	Adults with impaired glucose tolerance	Individualised counselling on diet, physical activity and weight reduction	Usual care	Mean 3.2 years	Diabetes incidence
Knowler et al. 2002 [9]	United States	3,234	Adults at high risk of diabetes	Intensive lifestyle curriculum targeting diet, exercise and weight loss	Placebo/usual lifestyle advice	Mean 2.8 years	Diabetes incidence
Kosaka et al. 2005 [20]	Japan	458	Men with impaired glucose tolerance	Lifestyle intervention aimed at achieving and maintaining ideal body weight	Standard intervention	4 years	Diabetes incidence
Ramachandran et al. 2006 [21]	India	531	Adults with impaired glucose tolerance	Lifestyle modification involving diet and physical activity advice	Control/standard advice	3 years	Diabetes incidence
Kawahara et al. 2008 [22]	Japan	426	Adults with impaired glucose tolerance	Two-day in-hospital diabetes education programme plus diabetes education/support every 3 months; diabetes education/support without hospitalisation was also assessed	General diabetes/IGT information without individual instruction or formal group counselling	3.1 years	Diabetes incidence and metabolic outcomes
Roumen et al. 2008 [23]	Netherlands	147	Adults with impaired glucose tolerance	Combined diet and physical activity lifestyle intervention	Usual care	3 years	Glycaemic outcomes and diabetes incidence
Yates et al. 2009 [14]	United Kingdom	103	Adults with impaired glucose tolerance	PREPARE structured education programme to promote walking, with or without pedometer support	Brief information/usual care	12 months	Glycaemic control and physical activity
Penn et al. 2009 [24]	United Kingdom	102	Adults with impaired glucose tolerance	Motivational interviewing targeting diet, physical activity and weight reduction	Usual care	3.1 years	Diabetes incidence
Saito et al. 2011 [25]	Japan	641	Overweight adults with impaired fasting glucose	Frequent lifestyle modification support	Less frequent intervention	36 months	Diabetes incidence
Sakane et al. 2011 [26]	Japan	304	Adults with impaired glucose tolerance	Primary-care lifestyle education using existing healthcare resources	Standard care	3 years	Diabetes incidence
Katula et al. 2013 [27]	United States	301	Adults with prediabetes	Community-based DPP-style lifestyle weight-loss programme	Enhanced usual care	24 months	Glycaemic and anthropometric outcomes
Davies et al. 2016 [15]	United Kingdom	880	Adults with intermediate hyperglycemia	Group-based structured diabetes prevention education programme	Standard care	3 years	Diabetes incidence and secondary outcomes
Hu et al. 2017 [28]	China	434	Elderly adults with prediabetes	Structured synthetic lifestyle intervention including education, diet, physical activity and follow-up support	Usual care	12 months	Diabetes incidence and glycaemic outcomes
Aekplakorn et al. 2019 [29]	Thailand	1903	Adults with impaired glucose tolerance	Community-based lifestyle modification through structured participatory group activities	Usual care	24 months	Diabetes incidence
Wani et al. 2020 [30]	Saudi Arabia	300	Overweight/obese adults with prediabetes	DPP-style intensive lifestyle monitoring focused on food choices, physical activity and weight loss	General advice	12 months	Weight and glycaemic outcomes
Sampson et al. 2021 [31]	United Kingdom	1028	Adults with high-risk intermediate glycaemia	Group-based lifestyle intervention with or without lay volunteer support	Control	Up to 46 months	Diabetes incidence
Khunti et al. 2021 [32]	United Kingdom	1366	Adults with non-diabetic hyperglycaemia/high diabetes risk	Walking Away structured behavioural physical activity education with differing support levels	Control	48 months	Physical activity and metabolic outcomes

Participants were adults with prediabetes or related non-diabetic hyperglycaemic states, most commonly diagnosed using impaired glucose tolerance, impaired fasting glucose or elevated glycated haemoglobin. The interventions included structured dietary education, physical activity promotion, walking programmes, weight-loss targets, individualised counselling, group education, motivational interviewing, self-monitoring, follow-up contacts and reinforcement. Comparator groups received usual care, standard advice, brief lifestyle information, general advice, enhanced usual care or less intensive support.

Risk of Bias Assessment

Risk of bias was assessed for the effect of assignment to intervention using the Cochrane RoB 2 tool [17]. Judgments were made for the principal review-relevant result at the longest applicable follow-up. Diabetes incidence was assessed where it was reported as the principal outcome; otherwise, the principal objective glycaemic, anthropometric or physical-activity outcome was assessed. For the cluster-randomised trials [7,15,29], the additional domain concerning the timing of participant identification or recruitment relative to cluster allocation was evaluated, see Table 2.

**Table 2:** Risk of bias assessment of included studies *This additional domain applies only to cluster-randomised trials; N/A indicates an individually randomised trial.

Study	Randomisation process	Timing of participant identification or recruitment*	Deviations from intended intervention	Missing outcome data	Outcome measurement	Selection of reported result	Overall risk of bias
Pan et al. [7]	Some concerns (limited allocation concealment detail)	Low risk: participants with impaired glucose tolerance were identified before clinic allocation	Low risk	Some concerns: completeness and handling of missing six-year outcome data were insufficiently reported	Low risk	Some concerns (older trial; protocol/reporting details limited)	Some concerns
Tuomilehto et al. [8]	Low risk	N/A	Low risk	Low risk	Low risk	Low risk	Low risk
Knowler et al. [9]	Low risk	N/A	Low risk	Low risk	Low risk	Low risk	Low risk
Kosaka et al. [20]	Some concerns (sequence generation and allocation concealment not clearly described)	N/A	Low risk	Some concerns: incomplete information about missing outcomes and their handling	Low risk	Some concerns (protocol/reporting details limited)	Some concerns
Ramachandran et al. [21]	Some concerns (allocation concealment not clearly described)	N/A	Low risk	Some concerns (attrition during follow-up)	Low risk	Some concerns (multiple intervention arms; reporting details limited)	Some concerns
Kawahara et al. [22]	Low risk	N/A	Low risk	Low risk	Low risk	Some concerns (protocol/reporting details limited)	Some concerns
Roumen et al. [23]	Some concerns (allocation concealment details limited)	N/A	Low risk	High risk: the three-year result was based on 106 of the 147 randomised participants, using a completer analysis	Low risk	Some concerns (selective reporting cannot be excluded)	High Risk
Yates et al. [14]	Some concerns (allocation concealment details limited)	N/A	Low risk	Low risk	Low risk for the objective glycaemic and activity outcomes assessed	Some concerns (secondary outcome reporting limited)	Some concerns
Penn et al. [24]	Some concerns (allocation concealment details limited)	N/A	Low risk	Some concerns (attrition during follow-up)	Low risk	Some concerns (protocol/reporting details limited)	Some concerns
Saito et al. [25]	Low risk	N/A	Low risk	Low risk	Low risk	Low risk	Low risk
Sakane et al. [26]	Low risk	N/A	Some concerns: possible contamination or variation in intervention implementation could not be excluded	Some concerns (28% withdrawal before 3-year follow-up)	Low risk	Some concerns (some secondary outcome reporting details limited)	Some concerns
Katula et al. [27]	Low risk	N/A	Low risk	Low risk	Low risk	Low risk	Low risk
Davies et al. [15]	Low risk	Low risk: participants were screened and identified before practice allocation	Low risk	Some concerns (loss to follow-up possible)	Low risk	Some concerns (some secondary outcomes incompletely reported)	Some concerns
Hu et al. [28]	Low risk	N/A	Low risk	Low risk	Low risk	Some concerns (protocol/reporting details limited)	Some concerns
Aekplakorn et al. [29]	Some concerns (cluster randomisation and allocation details limited)	Some concerns: participants appear to have been recruited after primary-care units were allocated	Low risk	High risk: completion was substantially lower in the control group 627/873 (71.8%) than in the intervention group 919/1030 (89.2%)	Low risk	Some concerns (reporting details limited)	High Risk
Wani et al. [30]	Some concerns (sequence generation and allocation concealment details limited)	N/A	Low risk	Some concerns (attrition during follow-up)	Low risk	Some concerns (protocol/reporting details limited)	Some concerns
Sampson et al. [31]	Low risk	N/A	Low risk	Low risk	Low risk	Low risk	Low risk
Khunti et al. [32]	Low risk	N/A	Low risk	Low risk	Low risk: the primary outcome was accelerometer-measured steps per day	Some concerns (complex intervention with multiple outcomes)	Low risk

Six studies were judged to have low overall risk of bias [8,9,25,27,31,32], 10 had some concerns [7,14,15,20-22,24,26,28,30], and two were judged to have high risk of bias [23,29]. Roumen et al. [23] was judged to have high risk because the three-year result was based on a completer analysis involving only 106 of the 147 randomised participants, meaning that missing outcomes could plausibly have affected the estimated intervention effect. Aekplakorn et al. [29] was judged to have high risk because outcome completion differed substantially between the control and intervention groups, at 71.8% and 89.2%, respectively. This differential loss could have biased the estimated effect if participants’ probability of remaining in the study was associated with intervention engagement or diabetes risk.

Effects of Structured Education Programmes on Study Outcomes

Progression to Type 2 Diabetes Mellitus: Progression to type 2 diabetes mellitus was the most frequently reported primary or major outcome across the included studies. Overall, structured education programmes focused on diet, exercise or combined lifestyle modification were generally associated with a lower incidence of type 2 diabetes compared with usual care, standard advice or less intensive lifestyle intervention [7-9,15,20-26,28,29,31].

The strongest evidence came from landmark diabetes prevention trials. The Da Qing study, the Finnish Diabetes Prevention Study and the Diabetes Prevention Programme all reported substantial reductions in diabetes incidence among participants receiving structured lifestyle intervention compared with control groups [7-9]. Similar benefits were reported in several subsequent trials from Japan, India, the United Kingdom, the Netherlands, China and Thailand, although the magnitude and statistical certainty of effect varied across studies [20-26,28,29,31].

However, effects were not uniform across all trials. Some pragmatic or lower-intensity programmes reported more modest or statistically uncertain effects on diabetes incidence, particularly where intervention intensity was lower, follow-up was limited, or the comparator group received some lifestyle advice [15,26]. Other studies focused primarily on physical activity, glycaemic or anthropometric outcomes rather than diabetes incidence as the main outcome [14,27,30,32]. This variation suggests that the effectiveness of structured education may depend on intervention intensity, participant engagement, follow-up duration, fidelity of delivery and the degree of contrast between intervention and comparator groups.

Glycaemic Control: Several studies assessed glycaemic outcomes, including fasting plasma glucose, 2-hour plasma glucose, oral glucose tolerance test results or glycated haemoglobin. Overall, structured education was associated with favourable changes in glycaemic indices in several studies, although the size and consistency of these effects varied [8,9,14,15,22,23,27,28,30].

In trials where lifestyle education achieved meaningful changes in weight, dietary intake or physical activity, glycaemic outcomes tended to improve. Improvements were particularly evident in more intensive or structured interventions that included individualised counselling, behavioural goals and repeated follow-up contacts [8,9,23,27,28,30]. Programmes focused mainly on walking or physical activity reported improvements in activity-related or selected metabolic outcomes, although effects on glycaemic indices were sometimes modest or not sustained [14,32].

Weight and Anthropometric Outcomes: Weight reduction was an important secondary outcome in many included trials. Structured education programmes that incorporated dietary advice, physical activity targets and behavioural support generally produced greater weight loss or more favourable anthropometric changes than usual care or less intensive advice [8,9,15,22,24,27,28,30-32].

The Diabetes Prevention Programme and other lifestyle trials demonstrated that weight reduction was an important component of diabetes risk reduction [9,24,27]. Programmes based on the Diabetes Prevention Programme model or other intensive lifestyle approaches tended to achieve more consistent improvements in body weight than less intensive programmes. However, the degree of weight loss varied across studies and was influenced by intervention duration, frequency of contact, baseline body weight, participant adherence and the availability of follow-up support.

Cardiovascular Risk Markers: Cardiovascular risk markers were reported less consistently than diabetes incidence, glycaemic control and weight. Where reported, structured education programmes showed variable effects on blood pressure, lipid profile, waist circumference and other cardiometabolic risk markers [8,9,15,22,23,28,30,32].

Some studies reported improvements in selected cardiovascular risk markers alongside improvements in weight or glycaemic control, while others found limited or inconsistent changes. This variability may reflect differences in baseline cardiovascular risk, intervention intensity, follow-up duration, medication use, dietary adherence and physical activity response. Overall, the evidence suggests that structured education may improve some cardiovascular risk markers, but the findings were less consistent than for diabetes incidence and weight-related outcomes.

Narrative Synthesis and Effect Direction Plot

Findings were synthesised narratively according to the principal outcome domains of progression to type 2 diabetes mellitus, glycaemic control, weight and anthropometric outcomes, and cardiovascular risk markers [19,33]. An effect-direction plot was used to summarise whether findings favoured structured education, favoured the comparator, showed no clear difference or were not reported [34] (Table 3).

**Table 3:** Effect direction plot of included studies ▲, favours structured education; ▼, favours comparator; ◄►, no clear difference or inconsistent findings; ▲/◄►, findings partly favoured structured education but were mixed across measures or time points; NR, outcome not reported or insufficient information available to determine the direction of effect.

Study	Diabetes incidence	Glycaemic control	Weight/anthropometry	Cardiovascular risk markers	Overall direction
Pan et al. [7]	▲	NR	NR	NR	Favours structured education
Tuomilehto et al. [8]	▲	▲	▲	◄►	Favours structured education
Knowler et al. [9]	▲	▲	▲	◄►	Favours structured education
Kosaka et al. [20]	▲	NR	▲	NR	Favours structured education
Ramachandran et al. [21]	▲	NR	◄►	NR	Favours structured education
Kawahara et al. [22]	▲	▲	▲	▲/◄►	Favours structured education
Roumen et al. [23]	▲	▲	▲/◄►	◄►	Favours structured education
Yates et al. [14]	NR	▲/◄►	NR	NR	Favours structured education for selected outcomes
Penn et al. [24]	▲	NR	▲	NR	Favours structured education
Saito et al. [25]	▲	NR	▲	NR	Favours structured education
Sakane et al. [26]	▲/◄►	NR	▲/◄►	NR	Favours structured education
Katula et al. [27]	NR	▲	▲	NR	Favours structured education
Davies et al. [15]	▲/◄►	▲/◄►	▲	◄►	Favours structured education for selected outcomes
Hu et al. [28]	▲	▲	▲	◄►	Favours structured education
Aekplakorn et al. [29]	▲	NR	▲	NR	Favours structured education
Wani et al. [30]	NR	▲/◄►	▲	◄►	Favours structured education for selected outcomes
Sampson et al. [31]	▲	NR	▲	NR	Favours structured education
Khunti et al. [32]	NR	◄►	▲/◄►	◄►	Mixed effect

The direction of effect was most consistent for progression to type 2 diabetes mellitus. Of the 14 studies reporting diabetes incidence, 11 favoured structured education or structured lifestyle intervention, while three reported partly favourable or mixed findings [7-9,15,20-26,28,29,31]. Weight and anthropometric outcomes were reported by 15 studies; 11 favoured structured education and four reported mixed or unclear findings [8,9,15,20-31,32]. Glycaemic outcomes were reported by 10 studies: six favoured structured education, three reported partly favourable or mixed findings, and one showed no clear difference [8,9,14,15,22,23,27,28,30,32].

Cardiovascular risk markers were the least consistent outcome domain. Most studies reporting blood pressure, lipid profile, waist circumference or related cardiometabolic measures showed no clear or consistent difference between structured education and comparator groups [8,9,15,22,23,28,30,32].

Certainty of Evidence

All five GRADE domains; risk of bias, inconsistency, indirectness, imprecision and publication bias, were considered for each outcome. As narrative synthesis was prespecified from the outset, inconsistency was assessed by comparing the direction, magnitude and coherence of findings across studies. Imprecision was assessed qualitatively using participant numbers, event counts where applicable, and confidence intervals reported by the contributing trials. Indirectness and publication bias were also assessed qualitatively.

The certainty of evidence for progression to type 2 diabetes mellitus was judged to be moderate. The evidence was downgraded by one level for risk of bias because some contributing trials had some concerns or were at high risk of bias, principally in relation to the randomisation process, missing outcome data or selection of the reported result. Nevertheless, the direction of effect was generally consistent and favoured structured education compared with usual care, standard advice or less intensive lifestyle intervention [7-9,15,20-26,28,29,31].

The certainty of evidence for glycaemic control was judged to be low. The evidence was downgraded by one level for risk of bias and by one level for inconsistency. Some contributing studies had concerns relating to randomisation, missing outcome data or selection of the reported result, and findings varied across fasting plasma glucose, two-hour plasma glucose and glycated haemoglobin measurements and across follow-up periods [8,9,14,15,22,23,27,28,30,32].

The certainty of evidence for weight and anthropometric outcomes was judged to be moderate. The evidence was downgraded by one level for risk of bias because some contributing studies had some concerns or were at high risk of bias. However, the direction of effect generally favoured structured education programmes incorporating dietary advice, physical activity targets and behavioural support compared with usual care or less intensive lifestyle advice [8,9,15,20-32].

The certainty of evidence for cardiovascular risk markers was judged to be low. The evidence was downgraded by one level for risk of bias and by one level for inconsistency because some contributing studies had some concerns or were at high risk of bias, and the reported effects on blood pressure, lipid profile and other cardiometabolic outcomes varied across studies [8,9,15,22,23,28,30,32].

Overall, moderate-certainty evidence indicated that structured education programmes focusing on diet and/or physical activity were associated with a lower risk of progression from prediabetes to type 2 diabetes mellitus and generally favourable weight and anthropometric outcomes compared with usual care or less intensive lifestyle advice. Low-certainty evidence suggested possible improvement in some measures of glycaemic control, whereas no clear or consistent effect was demonstrated for cardiovascular risk markers. These findings highlight structured education as a potentially valuable approach to delivering lifestyle support while recognising variation in programme content, intensity and delivery.

Discussion

This systematic review found that structured education programmes on diet and exercise reduce progression from prediabetes to type 2 diabetes mellitus and improve glycaemic and weight-related outcomes among adults with prediabetes. The direction of effect was most consistent for progression to type 2 diabetes, while findings for cardiovascular risk markers were less consistent. These findings support the role of structured education as an important component of diabetes prevention, particularly when programmes combine dietary advice, physical activity promotion, behavioural goal-setting and follow-up reinforcement.

The findings of this review are consistent with the Cochrane review by Hemmingsen et al. [10], which reported that combined diet and physical activity interventions can prevent or delay type 2 diabetes among people at increased risk. However, the present review differs from that broader review because it specifically focused on structured education, structured counselling and structured behavioural support rather than lifestyle intervention in general. This distinction is important because structured education provides a planned and organised approach to behaviour change, whereas brief or general lifestyle advice may not provide sufficient support for sustained dietary and physical activity modification.

Our findings also agree with the systematic review and network meta-analysis by Galaviz et al. [11], which showed that real-world lifestyle modification strategies can reduce diabetes incidence and promote weight loss. Similar to that review, the present review found that effects varied across studies, suggesting that programme design, intensity and delivery context influence effectiveness. However, while Galaviz et al. [11] focused on the real-world impact of lifestyle modification strategies more broadly, our review specifically examined whether structured education programmes on diet and exercise were more effective than usual care, standard advice or less intensive lifestyle support.

The findings also align with the long-term evidence summarised by Haw et al. [35], who reported that lifestyle modification strategies reduce diabetes incidence and have more sustained effects than medication-based prevention, although the benefits decline over time. This is relevant to the present review because several included interventions showed favourable effects during active follow-up, but intervention durability remains an important concern. Structured education programmes may therefore require ongoing reinforcement, maintenance sessions or long-term follow-up to preserve behavioural change and prevent attenuation of benefit over time.

The importance of intervention intensity observed in this review is also supported by Dunkley et al. [36], who found that pragmatic diabetes prevention programmes were effective in real-world settings, but that effectiveness varied substantially between programmes. Their review suggested that better adherence to guideline recommendations was associated with greater programme effectiveness. This supports the finding in the present review that programmes with repeated contact, goal-setting, dietary modification, physical activity targets and behavioural reinforcement tended to produce more favourable outcomes than lower-intensity interventions.

Similarly, Balk et al. [37] reported that combined diet and physical activity promotion programmes were effective in decreasing diabetes incidence and improving cardiometabolic risk factors among people at increased risk. Their findings support the overall conclusion of this review that structured education programmes combining dietary and physical activity components are beneficial for diabetes prevention. However, the present review found that effects on cardiovascular risk markers were less consistent than effects on diabetes incidence and weight-related outcomes. This may be because cardiovascular markers are influenced by additional factors, including baseline cardiovascular risk, medication use, adherence, intervention duration and the degree of weight loss achieved.

Weight reduction appeared to be an important pathway through which structured education reduced diabetes risk. This is consistent with Haw et al. [35], who reported that weight loss was a key factor associated with reduced progression to diabetes, and with Dunkley et al. [36], who found that lifestyle interventions in real-world settings produced significant weight loss. In the present review, studies that incorporated dietary targets, physical activity goals and behavioural support generally reported more favourable weight and anthropometric outcomes. This supports the view that structured education should move beyond information delivery alone and include practical strategies for weight management, self-monitoring and sustained behaviour change.

The present review also has implications for implementation. The economic review by Li et al. [38] found that diet and physical activity promotion programmes for diabetes prevention are cost-effective among people at increased risk. This strengthens the public health relevance of the present findings because structured education programmes may offer a scalable approach to diabetes prevention, particularly in primary care and community settings. However, cost-effectiveness is likely to depend on programme intensity, staffing, delivery format, participant retention and long-term maintenance of benefit.

This review adds to previous evidence by focusing specifically on structured education programmes for adults with prediabetes. Previous reviews have established that lifestyle modification reduces diabetes risk, but this review highlights that the method of delivery matters. Programmes that provide structured content, behavioural goals, follow-up support and reinforcement appear more useful than brief or unstructured advice. This is clinically important because patients with prediabetes are often advised to improve diet and exercise, but advice alone may not be enough to achieve sustained behaviour change.

The review has several strengths. It focused on randomised and cluster-randomised controlled trials, included studies from different countries and healthcare settings, and assessed risk of bias and certainty of evidence. The use of narrative synthesis and an effect direction plot allowed transparent presentation of findings despite heterogeneity in intervention content, delivery mode, follow-up duration and outcome reporting. The review also distinguished structured education from general lifestyle advice, which is important for clinical practice and service design.

There are also limitations. First, the included studies varied in diagnostic criteria for prediabetes, intervention intensity, comparator groups, delivery personnel, follow-up duration and outcome measures. This heterogeneity prevented statistical pooling and limited direct comparison between studies. Second, some trials had limited reporting of allocation concealment, intervention fidelity or secondary outcomes. Third, blinding of participants and personnel was generally not feasible because the interventions were educational and behavioural. Fourth, cardiovascular risk markers were reported less consistently than diabetes incidence, glycaemic control and weight-related outcomes. Finally, only English-language studies were included, which may have introduced language bias.

## Conclusions

The findings support the use of structured education programmes as part of diabetes prevention for adults with prediabetes. In clinical practice, lifestyle advice should be delivered through organised programmes that include education, individualised goal-setting, dietary and physical activity targets, behavioural support and follow-up reinforcement. Future research should identify the most effective and scalable components of structured education, examine long-term maintenance strategies, evaluate cost-effectiveness in different health systems and improve reporting of cardiovascular and implementation outcomes.

Overall, this review shows that structured education programmes on diet and exercise reduce progression from prediabetes to type 2 diabetes and improve glycaemic and weight-related outcomes. These findings are consistent with previous systematic reviews of lifestyle intervention, real-world diabetes prevention programmes and combined diet and physical activity promotion programmes, while adding specific evidence on the value of structured education as a delivery approach.
